# An introduction to phase ordering in scalar active matter

**DOI:** 10.1140/epjs/s11734-024-01273-5

**Published:** 2024-08-05

**Authors:** Laura Meissner, Julia M. Yeomans

**Affiliations:** The Rudolf Peierls Centre for Theoretical Physics, Clarendon Laboratory, Parks Road, Oxford, OX1 3PU UK

## Abstract

These notes provide an introduction to phase ordering in dry, scalar active matter. We first briefly review Model A and Model B, the long-standing continuum descriptions of ordering in systems with a non-conserved and conserved scalar order parameter. We then contrast different ways in which the field theories can be extended so that the phase ordering persists, but in systems that are active and do not reach thermodynamic equilibrium. The active models allow a wide range of dynamical steady states not seen in their passive counterparts. These include microphase separation, active foams and travelling density bands.

## Introduction

The individual particles that make up an active system take energy from their surroundings and use this to do work [18]. The continuous flow of energy means that active matter permanently remains out of thermodynamic equilibrium and can result in active particles having intricate, pattern-forming, collective motion. Examples range across scales: from the complex flight patterns of starling murmurations, to the co-operative motion of dense bacterial colonies and epithelial cell sheets, to the organisation of intra-cellular actin and microtubules, driven by motor proteins.

To understand such complex systems it is helpful to start with simpler examples and to study how the well-understood properties of equilibrium systems can be generalised to the active case. Here we summarise recent results taking this approach for phase ordering. Phase ordering is well understood in passive systems, those that relax to an equilibrium described by a free energy. Examples are the well-studied liquid–gas phase transition, ordering in a binary alloy or the transition to ferromagnetism [20]. The extent to which new physics should be implicated in describing phase ordering in active materials, for example the propensity of cells to sort into different cell-types [17] and the formation of biomolecular condensates within cells [3], remains an open question.

The aim of these notes is to provide a first introduction to phase ordering in a simple class of active matter, dry models described by a scalar order parameter. We first summarise the canonical equations that describe phase ordering in passive systems and then how these can be extended to include active terms. Despite the simplicity of the governing equations this uncovers a wide range of new physics. For those interested in reading further, reference [9] is an excellent, more detailed review.

## Field theory models for passive systems

**Fig. 1:**
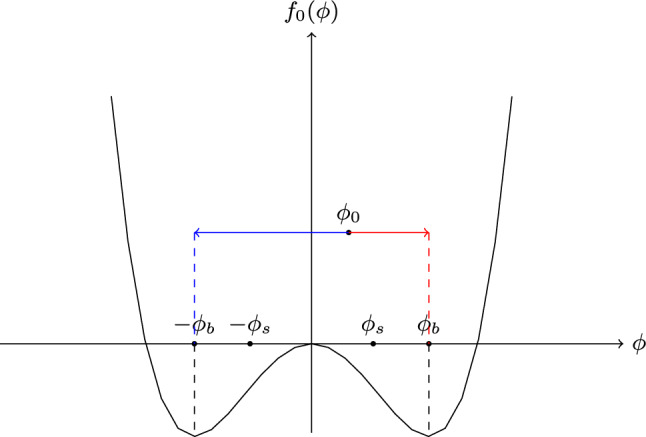
Free energy density $f_0(\phi )$ below the critical point. A system with initial uniform $\phi _0$ which lies between the binodal values $\pm \phi _b$ can separate into two phases to minimise the total free energy. The separation can happen either through spinodal decomposition if $|\phi _0|<\phi _s$ or through nucleation and growth if $\phi _b>|\phi _0|>\phi _s$

The scalar field models for active systems that we shall focus our attention on are extensions of equilibrium mean-field theories, which usually adopt the framework of a Ginzburg–Landau free energy expansion in an order parameter $\phi$. The free energy functional can be written

1 $$\begin{aligned} \mathcal{F}(\phi )= & {} \int \left\{ f_0(\phi )+\frac{\kappa }{2}(\nabla \phi )^2\right\} d\textbf{r}, \nonumber \\ f_0(\phi )= & {} \frac{a}{2} \phi ^2 + \frac{b}{4} \phi ^4, \end{aligned}$$

where $a<0$, $b>0$, $\kappa >0$ are constant parameters. These parameter constraints apply below the critical point, where phase separation can occur due to the double-well shape of $f_0(\phi )$ shown in Fig. 1. A linear term in Eq. (1) only contributes as a constant in the chemical potential and any cubic term can be removed by appropriate rescaling of $\phi$, therefore both of these have been omitted in $f_0(\phi )$. The thermodynamic chemical potential takes the form

2 $$\begin{aligned} \mu = \frac{\delta \mathcal{F}}{\delta \phi } = a \phi + b \phi ^3 - \kappa \nabla ^2 \phi , \end{aligned}$$

and it governs the temporal evolution of the order parameter.

Unless stated otherwise, we will assume $-a=b=\kappa =1$ for simplicity. We will investigate various active field models of dry scalar matter, meaning that the order parameter $\phi$ will represent a rescaled density field and the evolution of $\phi$ will not be coupled to momentum conservation as it would be in a hydrodynamic model [4, 9, 30]. The focus will mainly be on single-component systems in which phase separation occurs when dense and dilute domains form with clear interfaces. We will however also mention mixtures of interacting species, as they can result in new and interesting patterns.

To set the stage, we now introduce two simple canonical models used to study phase ordering in passive matter [19] – Model A and Model B.

### Model A

Model A describes the evolution of the order parameter in a system with no conservation laws, for example if chemical reactions [1, 21] or birth-death processes are present [7]. The dynamics is governed by the equation

3 $$\begin{aligned} \frac{\partial \phi }{\partial t} = -M \mu +\eta ({\textbf{r}},t) \end{aligned}$$

where *M* is a mobility coefficient that describes the relaxation rate of $\phi$ and $\eta$ is the noise, which is related to the mobility by the fluctuation-dissipation theorem

4 $$\begin{aligned} \langle \eta ({\textbf{r}},t) \eta ({\textbf{r}}^\prime ,t^\prime ) \rangle =2 {k}_B T M \delta (\textbf{r} - \textbf{r}^\prime ) \delta (t-t^\prime ). \end{aligned}$$

Within the context of these models the simplification is made to treat the mobility *M* as a constant. A more precise approach would be to recognise its dependence on $\phi$ but this would result in a multiplicative noise rather than an additive one which is significantly more difficult to deal with. This simple model describes relaxation to a target state for which the chemical potential, equation (2) is constant.

### Model B

A different category of systems is one in which the order parameter is conserved. To impose this constraint one can write the evolution of $\phi$ as a continuity equation

5 $$\begin{aligned} \frac{\partial \phi }{\partial t} + \nabla \cdot {\textbf{J}}=0 \end{aligned}$$

with a current ${\textbf{J}}$ of the form

6 $$\begin{aligned} {\textbf{J}}=-M \nabla \mu + \varvec{\eta } \end{aligned}$$

where *M* is the mobility and $\varvec{\eta}$ represents the noise contribution to the current and can again be related to *M* via the fluctuation-dissipation theorem. Due to the conservation law imposed on this model, the dynamics is not relaxational but rather a diffusive process, which can be seen more clearly by recasting the evolution equation as

7 $$\begin{aligned} \frac{\partial \phi }{\partial t} = M \nabla ^2 \mu + {\eta} \end{aligned}$$

where $\eta = \nabla \cdot \varvec{\eta}$. Model B, even in this passive form, already allows for bulk phase separation below the critical point. The double well shape of the free energy makes it possible for a system with initial uniform $\phi _0$ which lies between the binodal values $\pm \phi _b$ to lower its total free energy by separating into a denser and a more dilute phase, while retaining its overall conserved density as depicted in Fig. 1.

This process can occur either through nucleation and growth or spinodal decomposition. During nucleation and growth, nuclei appear locally and grow due to noise until a critical radius is reached, above which growth continues spontaneously. Spinodal decomposition on the other hand starts with small perturbations of the homogenous state which grow because of the instability of the free energy to ordering for $\mid \phi _0\mid <\mid \phi _s\mid$ where the spinodal density $\phi _s$ corresponds to zero curvature in $f_0(\phi )$.

In the later stages of phase separation, there are several processes that can cause domains of one phase to grow. This can happen for example through the collision and coalescence of individual droplets or through Ostwald ripening. Ostwald ripening occurs because there is a difference in pressure between droplets of different sizes. As the radius of curvature of a droplet increases, the pressure at the interface decreases as described by the Kelvin equation [16]. This can also be interpreted by considering the Laplace pressure jump across the interface of a droplet with radius *R* due to surface tension $\gamma$, which is

8 $$\begin{aligned} \Delta P \propto \gamma /R. \end{aligned}$$

This pressure, or equivalently chemical potential difference, drives flows from smaller to larger droplets eventually leading to bulk phase separation [8, 10, 11, 28, 32]. As we will see below, it is possible to slightly modify Model B in such a way, that Ostwald ripening can be reversed [14, 31].

## Field theory models of dry active matter

Now that we have established the simplest canonical mean-field models based on the Landau-Ginzburg free energy expansion in the chosen order parameter, we would like to extend these models to describe non-equilibrium systems which can break time-reversal symmetry or violate detailed balance. There are several routes one can choose for this purpose and the choice should be informed by the underlying physical, chemical, and biological dynamics.

One commonly used approach starts with the observation that active systems are out of thermodynamic equilibrium and therefore do not in general correspond to the minimum of a free energy. Therefore the chemical potential $\mu$ appearing in the evolution equations of active models can include additional contributions which cannot be derived from a free energy $\mathcal{F}$. These terms can then be considered to represent the ‘active’ contribution to the evolution of the order parameter [10, 31, 32]. It is common to use the Landau-Ginzburg free energy functional described in section 2 for the passive part of the chemical potential, and then to add terms of lowest possible order:

9 $$\begin{aligned} \mu = a \phi + b \phi ^3 - \kappa \nabla ^2 \phi + \mu _a, \end{aligned}$$

where $\mu _a$ includes all explicitly active contributions.

In an alternative approach the dynamics of the system is divided into sectors representing different types of processes within the system, and equilibrium-like free energies are constructed for each of these sectors. Detailed balance is then violated by allowing these free energies to differ, meaning that there is no global free energy for the entire system, and therefore that it is out of equilibrium.

Lastly, we will briefly consider mixtures of more than one particle species, for which a third approach can be utilised. Such systems can be kept out of equilibrium by choosing interactions between different species to be non-reciprocal.

### Introducing activity into the chemical potential

As summarised in section 2, canonical mean-field models for passive matter rely on defining a free energy, from which the chemical potential can be derived. The lowest order, simplest term that can represent activity in the chemical potential (9) is

10 $$\begin{aligned} \mu _a = \lambda \mid \nabla \phi \mid ^2, \end{aligned}$$

where $\lambda$ is a constant parameter. Such a contribution can be justified by explicit coarse-graining of the motion of self-propelled spherical particles or Active Brownian Particles [9, 27], where it arises as a consequence of collisions. From a physical perspective, this means that the velocity field is no longer a simple function of the density but a functional dependent on the density distribution within a persistence radius around a given position.

This coarse-graining procedure suggests that in systems where particles slow down as a result of higher density, we should expect $\lambda <0$. The evolution equations for the order parameter are however symmetric under $\lambda ,\phi \rightarrow -\lambda ,-\phi$ so using a $\lambda >0$ should not change the phase separation dynamics, only reverse the role of the phases. We will now consider the consequences of this active term for the specific cases of non-conserved and conserved order parameters.

### Active model A

First we turn our attention to Model A, equation (3), which describes the evolution of a non-conserved order parameter. Adding the activity term (10), leads to an equation of motion of the form

11 $$\begin{aligned} \dot{\phi } = \underbrace{\phi - \phi ^3}_A + \underbrace{\nabla ^2 \phi }_B - \underbrace{\lambda \mid \nabla \phi \mid ^2}_C, \end{aligned}$$

termed Active Model A [12] where we neglect noise for simplicity.

The RHS of this equation can be divided into three parts which we denote by *A*, *B*, *C*. *A* is a third-order polynomial with two stable fixed points at $\phi =\pm 1$ meaning that any fluctuation from a state with $\phi =0$ will grow to $+1$ or $-1$ so the final state would simply be an amplification of the initial conditions, without any clustering, as there is no gradient-driven dynamics yet.

Adding part B introduces diffusivity allowing clusters of the two phases to form. However eventually, because no conservation laws apply, the diffusive term, which acts on boundaries between phases, will lead to the eradication of one of the phases, leaving the system in a uniform state. If, however, the diffusivity dominates over the bulk terms, it will quickly restore any initial perturbations and the system will settle in the remixed $\phi =0$ state.

**Fig. 2:**
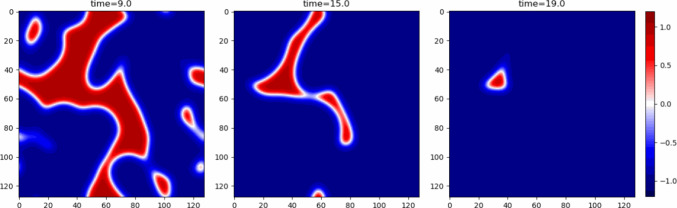
Snapshots from a simulation of Active Model A with activity parameter $\lambda =1$ showing the system evolving to a homogeneous steady state with $\phi =-1$

For the active term, *C*, the sign of $\dot{\phi }$ depends on the sign of $\lambda$. If $\lambda <0$ any gradients will lead to an increase of the order parameter and the uniform $\phi =+1$ configuration will be the stationary stable state. Analogously for $\lambda >0$ the stable state will be $\phi =-1$. However, unlike in the case of diffusivity, the system reaches the uniform state directly rather than through a stage of phase separation. A typical evolution of Active Model A is shown in Fig. 2.

These arguments show that adding the lowest order active term to the chemical potential derived from the Landau-Ginzburg free energy does not qualitatively change the behaviour of Model A. Depending, however, on the relative sizes of the contributions in equation (11) activity can accelerate or decelerate the phase ordering.

### Active model B

We next generalise Model B, which describes ordering in a system with a conserved order parameter to Active Model B [9, 32]. Putting together equation (6) for the order parameter current and the active chemical potential defined by Eqs. (9) and 10) gives

12 $$\begin{aligned} {\textbf{J}} = \underbrace{(1 - 3\phi ^2)\nabla \phi }_A + \underbrace{\nabla \nabla ^2 \phi }_B - \underbrace{\lambda \nabla \mid \nabla \phi \mid ^2}_C. \end{aligned}$$

A characteristic feature of this model is that even with activity the current can be written as the gradient of a chemical potential and therefore the model is curl free. This implies that there can be no circulating net flows which would allow for a steady state with non-zero currents. This issue can be addressed by introducing another active term of the same order and we will return to this point later in Sect. 3.4 [9, 10, 31].

We again divide the expression into three terms to investigate the role of each in the dynamics of the system. The form of part A shows that the current depends on the direction of the gradient of the order parameter and also on the absolute value of $\phi$. As long as $\mid \phi \mid <1/\sqrt{3}$, the current flows in the direction of the gradient, therefore amplifying the spatial variation of $\phi$. Note that this corresponds to the region between the spinodals, $d^2 f_0/d \phi ^2<0$, where the uniform state is linearly unstable. Once $\mid \phi \mid >1/\sqrt{3}$ the current reverses direction to locally smooth out the density distribution.

The gradient term B can be derived from a free energy functional and within that free energy description it acts as a surface tension penalising boundaries. In terms of the current material flows away from dense regions into dilute regions.

C is the active term. Assuming $\lambda <0$ this new contribution to the current acts to drive material towards the steepest gradients of $\phi$ from more homogeneous regions. An argument for this is that in regions of local maxima of $\phi$ this term vanishes, since the individual particles slow down due to overcrowding. In places where $\phi$ has a minimum, there is very little material to contribute to the current and therefore it also tends to zero.

The dynamics of Active Model B have been studied numerically [32]. It was found that the macroscopic behaviour does not differ qualitatively from its passive counterpart.

**Fig. 3:**
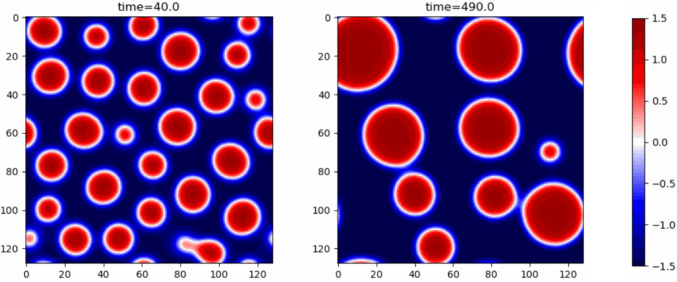
Snapshots from a simulation of active model B with activity parameter $\lambda =-2$ and mean global density $\overline{\phi }=-0.4$ showing Ostwald ripening

As a consequence of the activity the thermodynamic pressure calculated from the free energy is not equal in the two phases. It is possible to show that for $\lambda <0$ and a dense droplet in dilute surroundings (or dilute bubble in dense surroundings when $\lambda >0$) the Laplace pressure across an interface can be balanced by the jump in the active pressure at a finite radius $R \propto 1/ \mid \lambda \mid$. This would suggest that Active Model B also allows for microphase separation, however, this solution turns out to be unstable to perturbations, so that slightly larger droplets grow while smaller droplets shrink, leading again to bulk phase separation by Ostwald ripening (Fig. 3).

### Active model B+

Active model B does not allow for any stable microscopic pattern formation or circulating currents, but such features have been reported in experiments and simulations [5, 6, 27, 29]. To allow for these more interesting phenomena one can introduce a second active term into Model B leading to model B$+$ [31]:

13 $$\begin{aligned} \textbf{J}= & {} -\nabla \mu + \zeta (\nabla ^2\phi )\nabla \phi \nonumber \\= & {} \underbrace{(1-3\phi ^2)\nabla \phi }_\text {A} +\underbrace{\nabla \nabla ^2\phi }_\text {B} -\underbrace{\lambda \nabla |\nabla \phi |^2}_\text {C} + \underbrace{\zeta (\nabla ^2\phi )\nabla \phi }_\text {D}. \end{aligned}$$

C and D are the only possible active terms to this order in $\nabla$ and $\phi$. This expression can be justified by explicit coarse-graining of the microscopic motion of active Ornstein–Uhlenbeck particles that are subject to an active force which consists of exponentially correlated noise with a finite persistence time, and a symmetric, weak, long-range two-body interaction $\nabla U(|\mathbf {r_i-r_j}|)$ [31]. The interaction between particles leads to the new contribution to the current in the form of term D. The coarse-grained model implies that the new activity coefficient $\zeta$ depends on the specific form of *U*, the persistence time of the active force and the density-dependent particle speed. Within the canonical form of Model B, these dependencies are suppressed and $\zeta$ is treated as a constant parameter. It remains the case that the dynamics are invariant under the transformation $(\phi ,\lambda ,\zeta )\rightarrow (-\phi ,-\lambda ,-\zeta )$, which means that a change in sign of both activity parameters implies an exchange of roles between the dense and dilute phases.

In one dimension both active terms C and D take on precisely the same form

14 $$\begin{aligned} \underbrace{-\lambda \big ((\phi ')^2\big )'}_\text {C} + \underbrace{\zeta \phi ''\phi '}_{D} = (\zeta -2\lambda )\phi '\phi '' \end{aligned}$$

where the prime superscript denotes the spatial derivative. One, therefore, needs to move into two dimensions to recognize the difference in the dynamical behaviour of $\phi$ introduced by term D. The direction of the new current depends on the gradient of $\phi$ and the sign of $\nabla ^2\phi$. This implies that in dense regions the current flows with decreasing magnitude towards dilute regions vanishing at points of inflection. In dilute regions on the other hand, when $\nabla ^2\phi >0$ current moves along the gradient towards denser regions. More importantly, the resulting current no longer has to be curl-free allowing for circulating dynamics.

**Fig. 4:**
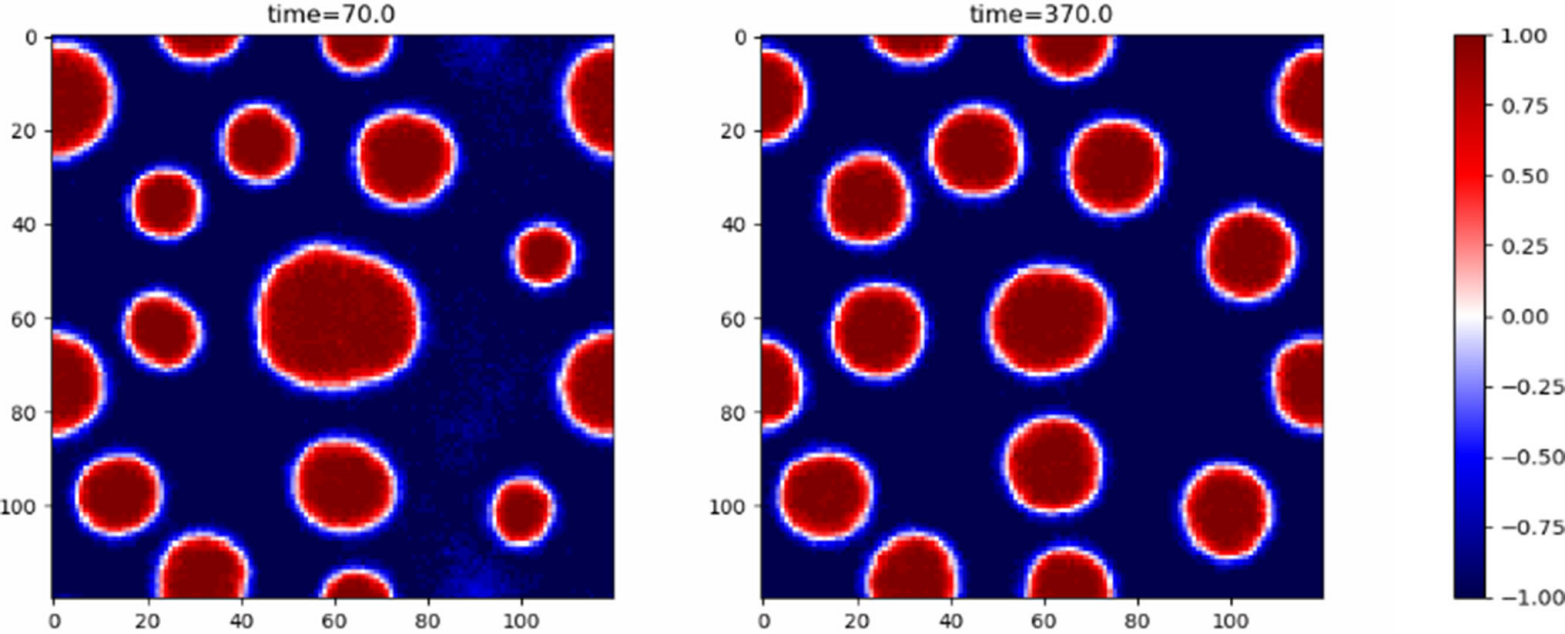
Snapshots from a simulation of Active Model B+ with activity parameters $\lambda =-1.5,\zeta =-2$ and mean global density $\overline{\phi }=-0.5$ showing reversed Ostwald ripening. Larger droplets shrink while smaller droplets grow until all droplets have the same radius

Analytic considerations and simulations of Model B+ [14, 15, 31] have demonstrated the existence of new steady states. Firstly, one can consider spherical droplets or bubbles and their dynamics. It is useful to define a pseudo-tension $\sigma$, which determines the pseudo-pressure jump across curved interfaces in a way analogous to the Laplace pressure jump due to surface tension in equilibrium systems. This pseudo-tension depends on all three gradient parameters $\kappa ,\lambda ,\zeta$ and, in particular, can take on a negative sign, resulting in the reversal of Ostwald ripening. (Note that the pseudo-tension is not directly related to the mechanical tension and does not cause interfaces to become unstable when it takes a negative value [31].)

The reason for this reversal can be understood in terms of the coexisting phases $\phi _{\pm }$ and their stationary state dependence on the droplet radius $R_d$. In Model B, the density around small droplets exceeds the density around large droplets, causing a flow of material towards larger droplets i.e. Ostwald ripening. Within the parameter space of $(\lambda ,\zeta )$ however there are regions where the density around larger droplets exceeds the density around smaller ones inducing a flow in the opposite direction. This results in a steady state of dense droplets (vapour bubbles) of a fixed radius floating in a bath of the dilute phase (dense phase) as shown in Fig. 4.

Flat interfaces have also been studied in the context of Active Model B+ [14]. The capillary tension $\sigma _c$, that controls the evolution of height perturbations on the interface can become negative introducing instabilities. Assuming $\zeta >0$, in the dilute phase ($\phi _-$) diffusive currents at the interface are always stabilising, however on the dense side ($\phi _+$) they can become destabilising and if these currents dominate over the stabilising currents then fluctuations of the boundary will grow.

This instability can result in a bubbly phase, and also in a new type of microphase separated state, referred to as an active foam [14]. The bubbly phase induced by interface instabilities develops by individual bubbles detaching from the interface, dynamically changing shape and size, coalescing and dividing until a steady state is reached. This differs significantly from the reverse Ostwald ripening process in which bubbles are formed as a result of balancing nucleation, diffusive flow and coalescence. A further implication of this difference is that the steady-state bubble radius $R_b$ is not the same. This can be illustrated by the observation that in the case of nucleation and reverse Ostwald ripening $R_d\rightarrow \infty$ as we reduce the noise to zero stopping new bubbles from nucleating. This is however not the case for instabilities, where an initial perturbation is sufficient to induce a bubbly phase [14, 15].

**Fig. 5:**
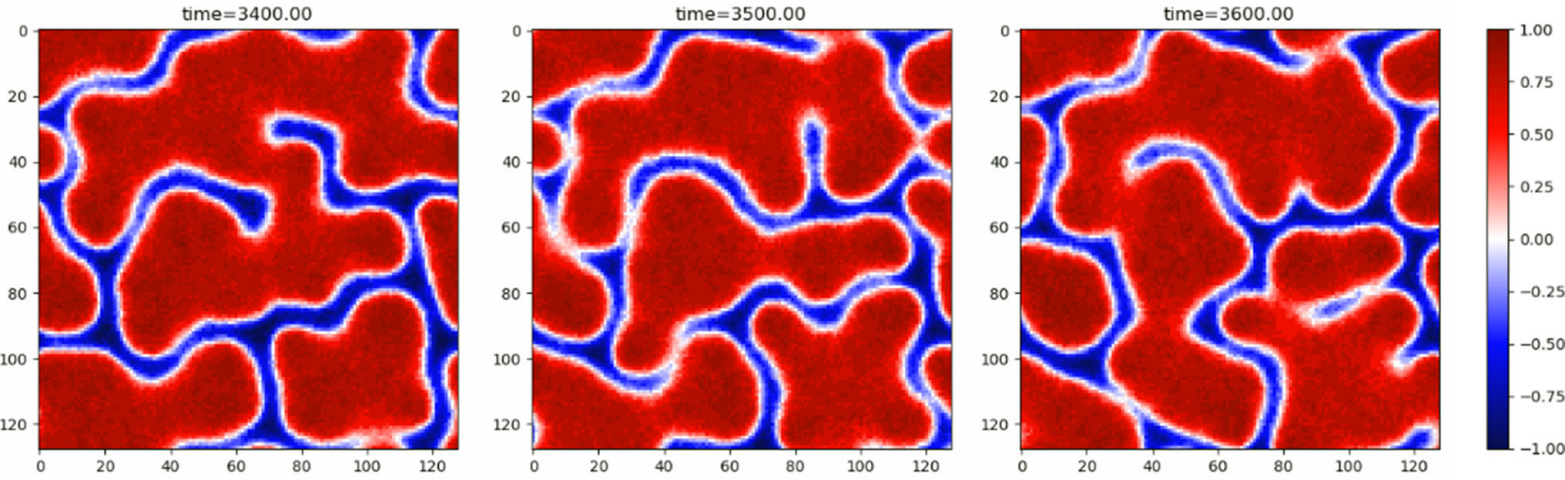
Snapshots from a simulation of Active Model B+ with activity parameters $\lambda =-2,\zeta =-2$ and mean global density $\overline{\phi }=0.4$ showing the active foam state

An active foam state forms when the amount of stabilising phase (dilute for $\zeta >0$) dominates over the destabilising phase (dense for $\zeta >0$) preventing the formation of bubbles. The instability causes interfaces to bend towards the stabilising phase. As a result, the destabilising phase forms long threads creating a dynamical web with connections continuously forming and breaking as shown in Fig. 5.

### Active model AB

It is possible to define dynamical systems that simultaneously undergo several processes some of which do, and some of which do not, conserve the density. An example is model AB [22–24] which is defined by the equations

15 $$\begin{aligned} \frac{\partial \phi }{\partial t}&= -\nabla \cdot \textbf{J} - M_A\mu _A + \eta _A, \nonumber \\ \textbf{J}&= -M_B\nabla \mu _B + \eta _B \end{aligned}$$

where the subscripts A,B distinguish processes that respectively do not, or do, conserve the density. To introduce time-reversal symmetry breaking one could choose $\mu _A,\mu _B$ to not be derivatives of free energies $F_A, F_B$, as for Active Model A, B and B+. However, an alternative and computationally simpler approach is to let $F_A\not = F_B$ with $\mu _A = \delta F_A/\delta \phi$ and $\mu _B = \delta F_B/\delta \phi$. This can be interpreted as moving the conservative and non-conservative parts of the dynamics out of equilibrium with each other but allowing both of these processes to reach equilibrium in isolation. Thus the system undergoes relaxational (A) and diffusive dynamics (B), but with the two processes being thermodynamically out of balance.

To describe relaxation the simplest choice for $\mu _A$ would be $\mu _A \propto (\phi -\phi _t)$ where $\phi _t$ is a target density towards which the system relaxes. More interesting phenomena are however possible when $\mu _A$ contains non-linearities [22]. Hence, the canonical choice is

16 $$\begin{aligned} \mu _A(\phi ) = A(\phi -\phi _t)(\phi -\phi _a), \end{aligned}$$

where without loss of generality we can assume $\phi _a<\phi _t$. Such a chemical potential defines two fixed points, one of which is stable ($\phi _t$) and one unstable ($\phi _a$).

The conservative sector is chosen to be described by the Landau-Ginzburg free energy, as for Model B, with the corresponding chemical potential

17 $$\begin{aligned} \mu _B(\phi ) = -\alpha \phi +\beta \phi ^2-\kappa \nabla ^2\phi . \end{aligned}$$

Combining these particular choices for $\mu _A$ and $\mu _B$, the final equations of Model AB, neglecting noise, become

18 $$\begin{aligned} \frac{\partial \phi }{\partial t}&= -\nabla \cdot {\textbf{J}} - AM_A(\phi -\phi _t)(\phi -\phi _a),\end{aligned}$$

19 $$\begin{aligned} \textbf{J}&= M_B\nabla (\alpha \phi -\beta \phi ^3+\kappa \nabla ^2\phi ). \end{aligned}$$

Similarly to Active Model B+, Model AB can support microphase separation in the form of bubbles, droplets or more elongated domains, depending on the target density set by $\phi _t$, as shown in Fig. 5. This occurs when the target density satisfies $|\phi _t|<\phi _s$, where $\pm \phi _s$ are the spinodals of $F_B$. The target density is then unstable due to diffusion, so the system can undergo spinodal decomposition. Model B dynamics will work towards bulk phase separation by driving currents from dilute to dense regions, Model A dynamics on the other hand decreases the density if $\phi >\phi _t$ and increases it otherwise, meaning that, broadly speaking, material is created in dilute regions, transported by diffusion into dense regions and then destroyed in the dense regions. The balance between these processes results in a steady state of microphase separation. Systems with $\phi _t$ lying outside the spinodal but within the binodal region also undergo microphase separation, but through nucleation and growth rather than spinodal decomposition [22, 23].

The specific patterns that can form from Model AB dynamics also depend on the initial conditions. Instead of starting with a uniform state and small random fluctuations, one can start from a dense droplet in a dilute bath. Within a small subspace of the parameter space, it is then possible to obtain a pattern of concentric rings formed via spinodal decomposition. At a fine-tuned level of noise, these rings can break up forming a lamellar pattern of nearly straight, parallel stripes with bends which can be interpreted as a manifestation of the Helfrich-Hurault instability [22].

If $\mu _a$ is non-linear the interplay between diffusive and relaxational processes can also lead to a new type of dynamical behaviour where the system oscillates between a phase-separated state and the uniform state. Consider a system which is originally in a uniform state with a mean global order parameter between the binodal and spinodal values. $\phi$ has no spatial dependence, therefore the diffusive current vanishes and the density increases globally towards $\phi _t$, driven by the non-conservative dynamics. Once the spinodal density has been reached the system becomes unstable locally and can undergo spinodal decomposition, separating into domains of densities $~\pm \phi _b$. The model parameters can be chosen so that these densities correspond to values where the Model A dynamics decreases the global order parameter. Thus the system can be driven to densities where the phase separated state is no longer stabilised by the diffusive dynamics and remixing occurs completing the cycle.

### Non-reciprocal interactions

Finally we mention another possible way to introduce activity into canonical field models such as Model A and Model B. When describing a system composed of two interacting species, S1 and S2 say, the system can be forced out of equilibrium by breaking detailed balance, i.e. by introducing non-reciprocal interactions such that S1 particles respond to S2 particles in a different way than S2 particles respond to those of species S1. Typical equations of motion are

20 $$\begin{aligned} \frac{\partial \phi _i}{\partial t}&= -\nabla \cdot \textbf{J}_i, \, \end{aligned}$$

21 $$\begin{aligned} \textbf{J}_i&= -\nabla \mu _i^{0} + \sum _j \alpha _{ij}\nabla \phi _j, \end{aligned}$$

where the subscripts *i*, *j* denote any number of different species, $\mu _i^0$ are the corresponding equilibrium chemical potentials and $\alpha _{ij}$ are interaction parameters such that $\alpha _{ij}\not = \alpha _{ji}$, which is the condition for non-reciprocity. A common and simple approach is to choose $\alpha _{ij}$ to be an anti-symmetric matrix.

The possible steady states, depend on the strength of the non-reciprocal coupling, and include static bulk phase separation, travelling microphase-separated patterns [26] and oscillating structures [33]. Travelling patterns, which often occur in the form of self-propelled lamellar domains, are a manifestation of a non-reciprocal run-and-catch mechanism, whereby domains of S1 are attracted to domains of S2 and hence try to “chase” them, while domains of S2, which are repelled by S1 “run away”. This behaviour occurs at high activity when the non-reciprocal interactions dominate over passive dynamics. Another dynamic steady state that has been reported in recent work [33] is oscillating patterns comprising dense and dilute domains periodically merging and splitting. The emergence of this behaviour is most probably determined by the effect of initial conditions and noise on the same underlying dynamics as governs travelling patterns.

## Discussion

We have listed several strategies that can be employed to introduce activity into the simple field theories commonly used to describe phase ordering in systems with a scalar order parameter $\phi$. These include modifying the chemical potential which governs the evolution of $\phi$ by adding terms which cannot be derived from a free energy functional, introducing non-reciprocal interactions, or coupling conserved and non-conserved dynamics that relax to different equilibria. The models can be written down phenomenologically, in the spirit of a Landau expansion, or can often also be justified by coarse-graining the microscopic dynamics of an active particle model.

The resulting phase space can be complex with steady states that range from bulk phase separation to bubbles, droplets, elongated filaments and active foam patterns. Travelling bands and density oscillations have also been identified. The same pattern can often be reached via several routes including reversed Ostwald ripening, spinodal decomposition, and interface instabilities. Moreover the specific outcome can depend not only on parameter values, but also on initial conditions and the noise level.

The richness of this behaviour suggests interesting routes to phase ordering will be found in active systems with more complex order parameters, such as active nematics [13] or chiral active matter [25]. Active model H and related systems, where the order parameter is coupled to a velocity field, have also been studied recently [2, 30]. Phase ordering is common in biological systems, where it is commonly referred to as cell sorting [17]. Although this has traditionally been explained in terms of differential adhesion, a passive concept, it is reasonable to ask whether the activity of cells can also contribute to their propensity to sort.

## Data Availability

No Data associated in the manuscript.
